# Interaction of Fibromodulin and Myostatin to Regulate Skeletal Muscle Aging: An Opposite Regulation in Muscle Aging, Diabetes, and Intracellular Lipid Accumulation

**DOI:** 10.3390/cells10082083

**Published:** 2021-08-13

**Authors:** Eun Ju Lee, Syed Sayeed Ahmad, Jeong Ho Lim, Khurshid Ahmad, Sibhghatulla Shaikh, Yun-Sil Lee, Sang Joon Park, Jun O. Jin, Yong-Ho Lee, Inho Choi

**Affiliations:** 1 Department of Medical Biotechnology, Yeungnam University, Gyeongsan 38541, Korea; gorapadoc0315@hanmail.net (E.J.L.); sayeedahmad4@gmail.com (S.S.A.); lim2249@naver.com (J.H.L.); ahmadkhursheed2008@gmail.com (K.A.); sibhghat.88@gmail.com (S.S.); jinjo@yu.ac.kr (J.O.J.); 2 Research Institute of Cell Culture, Yeungnam University, Gyeongsan 38541, Korea; 3 Department of Molecular Genetics, School of Dentistry and Dental Research Institute, Seoul National University, Seoul 08826, Korea; yunlee@snu.ac.kr; 4 College of Veterinary Medicine, Kyungpook National University, Daegu 41566, Korea; psj26@knu.ac.kr; 5 Department of Biomedical Science, Daegu Catholic University, Gyeongsan 38430, Korea; ylee325@cu.ac.kr

**Keywords:** fibromodulin, myostatin, muscle aging, sarcopenia, skeletal muscle

## Abstract

The objective of this study was to investigate fibromodulin (FMOD) and myostatin (MSTN) gene expressions during skeletal muscle aging and to understand their involvements in this process. The expressions of genes related to muscle aging (Atrogin 1 and Glb1), diabetes (RAGE and CD163), and lipid accumulation (CD36 and PPARγ) and those of FMOD and MSTN were examined in CTX-injected, aged, MSTN^−/−^, and high-fat diet (HFD) mice and in C2C12 myoblasts treated with ceramide or grown under adipogenic conditions. Results from CTX-injected mice and gene knockdown experiments in C2C12 cells suggested the involvement of FMOD during muscle regeneration and myoblast proliferation and differentiation. Downregulation of the FMOD gene in MSTN^−/−^ mice, and MSTN upregulation and FMOD downregulation in FMOD and MSTN knockdown C2C12 cells, respectively, during their differentiation, suggested FMOD negatively regulates MSTN gene expression, and MSTN positively regulates FMOD gene expression. The results of our in vivo and in vitro experiments indicate FMOD inhibits muscle aging by negatively regulating MSTN gene expression or by suppressing the action of MSTN protein, and that MSTN promotes muscle aging by positively regulating the expressions of Atrogin1, CD36, and PPARγ genes in muscle.

## 1. Introduction

Skeletal muscle (SM) is the largest organ in the body, constitutes 30–40% of total body weight, and is important for maintaining posture and controlling blood glucose levels and body temperature [1,2,3]. SM contains a diverse population of muscle stem (or satellite) cells (MSCs), which are responsible for postnatal growth, muscle regeneration, and important for functional SM integrity and maintenance of muscle tissue [4,5]. The co-expressions of the paired box transcription factors (Pax3 and Pax7) and the expressions of myogenic regulatory factors like MYOD, MRF4 (muscle regulatory factor 4), Myf5, and myogenin (MYOG) are responsible for MSC progression [6,7]. The self-renewing property of MSCs preserves the stem cell population and affords several types of myogenic cells, which proliferate, differentiate, and fuse to form myofibers [8]. However, age-related muscle damage and extreme muscle mass loss are related to poor prognoses in patients with myopathy or muscular dystrophy [9].

Myostatin (MSTN) is a myokine that is mainly expressed in SM and belongs to the TGF-β superfamily [10]. MSTN is a well-known negative regulator of muscle growth and has been intensively studied since inactivation of the MSTN gene in mice and its mutation in cattle, sheep, or man were reported to accelerate muscle growth [10,11]. MSTN also plays vital regulatory roles during MSC proliferation and differentiation by hindering the transcription factors Pax7, MYOG, and MYOD [10,12,13]. The mature form of MSTN binds to its receptor, which, when activated, signals the downregulation of protein synthesis and upregulation of protein degradation [14]. MSTN is also highly expressed in adipose tissues and is viewed as a potential target in obesity and type 2 diabetes mellitus because of the prominent role it plays in insulin-mediated glucose disposal and in determining the metabolic rate of SM [15,16,17]. Furthermore, MSTN knockdown has been reported to enhance SM development significantly and to reduce intramuscular fat contents in wild-type animals [15,18,19,20,21].

Extracellular matrix (ECM) creates biochemical signals that regulate myogenesis [22], and integrins are the core receptors in the context of facilitating communication between cell surfaces and their microenvironments [23,24]. These receptors are composed of heterodimeric (alpha and beta) subunits that bind to various types of ECM ligands. Fibromodulin (FMOD) is a member of the proteoglycan family and assists ECM assembly and participates in myoblast differentiation by controlling the interaction between MSTN and type IIB activin receptor (ACVRIIB) [3,24,25]. At sites of injury, FMOD plays a key role in muscle regeneration by increasing the enrollment of MSCs, and in this background, FMOD maintains its transcriptional activity and overcomes the inhibitory effects of MSTN [26,27]. FMOD also plays critical roles during collagen fibrillogenesis, cell adhesion, in the modulation of cytokine activity, the suppression of tumor growth, and the inhibition of apoptosis [28,29,30]. Interestingly, FMOD up-regulation also promotes proliferation and migration of pancreatic stellate cells [31].

Muscle deterioration is associated with different types of catabolic diseases and with reductions in muscle mass, functional ability, and quality of life, and increases the risk of morbidity and mortality [14]. Atrogin-1 is a key E3 ubiquitin ligase involved in atrophy that is significantly expressed in SM during muscle atrophy. Muscle atrophy in mice lacking atrogin-1 is resistant to denervation-induced muscle atrophy. Furthermore, atrogin-1 knockdown inhibits muscle loss during fasting [32,33]. Gradual loss of SM mass and strength during aging is termed sarcopenia [34], and aging is characterized by accumulations of reactive oxygen species and DNA damage, mitochondrial dysfunction, impaired antioxidant defense, and changes in gene and non-coding RNA expressions [35]. Furthermore, aging negatively regulates brown adipocyte formation and function [36], and high intramyocellular lipid (IMCL) content is a characteristic metabolic feature in aged and obese SMs [37,38] and can play central roles in the development of muscle resistance to anabolic stimuli and the progressions of muscle atrophy in the obese and in patients with sarcopenia [38,39,40,41].

A previously reported study on gene expression profile during differentiation/transdifferentiation in MSC from muscle tissue and preadipocytes showed a correlation between intramuscular fat and adipocyte cells to regulate the formation of brown adipose tissue [42]. Another study reported that advanced glycation end product (AGE) upregulation was associated with myogenic marker gene downregulation and reduced myotube formation [43]. A promising relationship between FMOD and MSTN was reported in which FMOD was found to be a regulator of MSTN for proliferation and differentiation of MSCs [4,26]. FMOD is a component of ECM and transmits its signal to activate calcium channels via collagen type 1 alpha 1 (COL1α1) and integral membrane protein 2a (ITM2a), and thus, induces myogenic differentiation [44]. This study was conducted to explore the roles played by FMOD and MSTN and their inter-connection throughout the myogenic program and to investigate their effects during SM regeneration and aging.

## 2. Materials and Methods

### 2.1. Mouse Experiment

C57BL/6 male mice were purchased from Daehan Biolink (Dae-Jeon, Korea) and maintained four per cage in the temperature-controlled room under a 12 h light cycle. Animals were provided standard rodent chow containing 4.0% (wt/wt) total fat (Rodent NIH-31 Open Formula Auto; Zeigler Bros., Inc., Gardners, PA, USA) and water. All animal-related experiments complied with the guidelines issued by the Institutional Animal Care and Use Committee of Yeungnam University (YUMC-AEC2015-006). Gastrocnemius muscles (gas muscle) tissues were excised in 16, 26, and 104 (2 years) weeks old mice. To investigate FMOD and MSTN expression patterns during muscle regeneration, 100 µL of 100 nM cardiotoxin (CTX) was injected once into gas muscle and maintained for 3 or 7 days. All treatments were performed under i.p. Avertin anesthesia. Non-CTX and CTX-injected muscle tissues were collected for protein extraction or fixed for immunohistochemistry. MSTN knockout mice were provided by Lee’s Lab at Seoul National University [45]. Normal, MSTN^+/−^ (heterozygote), and MSTN^−/−^ (homozygote) gas muscle tissues were harvested from 6-week-old mice, fixed, and stored at −80 °C until required for analysis.

### 2.2. Cell Culture

Mouse C2C12 myoblast cells (Korean Cell Line Bank, Seoul, Korea) were cultured in growth media [DMEM (HyClone Laboratories, UT, USA) + 10 % FBS (fetal bovine serum, HyClone Laboratories) + 1% P/S (Penicillin/Streptomycin, Thermo Fisher Scientific, Waltham, MA, USA)] at 37 °C in 5% CO_2_ atmosphere. To induce myoblast differentiation, the growth medium was switched to differentiation medium [DMEM + 2% FBS + 1% P/S]. To investigate lipid accumulation, cells were cultured in adipogenic medium [10 µg /mL insulin (Sigma-Aldrich, St. Louis, MO, USA) + 1 µM dexamethasone (Sigma-Aldrich) + 0.5 mM 3-isobutyl-1-methylxanthine; IBMX (Sigma Aldrich)] for 2 days and then incubated with 10 µg/mL insulin for a further 2 days. To induce aging, myoblasts were cultured for 2 days in growth or differentiation media supplemented with 50 µM ceramide (Sigma Aldrich).

### 2.3. Metabolite Analysis

C2C12 cells were cultured in differentiation media supplemented with DMSO or 50 µM ceramide for 2 days and then culture media were collected. Concentrations of glucose, lactate, and NH_3_ in culture media were measured using appropriate detection reagents (Glucose, Lactate, and NH_3_ Bio HT, Roche Diagnostics, Indianapolis, IN, USA) and a Cedex Bioanalyzer (Roche Diagnostics).

### 2.4. MTT Assay

C2C12 cells were washed with DMEM and incubated with 0.5 mg/mL of MTT reagent (Sigma-Aldrich) for 1 h at 37 °C. DMSO was added to dissolve the formazan crystals formed and absorbance was measured at 540 nm using a Versa Max microplate reader (Tecan Group Ltd., Männedorf, Switzerland).

### 2.5. Galactosidase Staining

Galactosidase activities were assessed using an X-gal kit (Cell Signaling Technology, Danvers, MA, USA). Cells were washed with PBS, fixed with fixative solution, treated with β-galactosidase staining solution, incubated overnight in a dry incubator, washed with PBS, and observed under an optical microscope.

### 2.6. Oil Red O Staining

C2C12 cells were washed with PBS, fixed with 10% formaldehyde (Sigma-Aldrich) for 20 min, and incubated with Oil Red O solution [6:4 dilution of stock (3.5 mg/mL Oil Red O powder in 100% isopropanol)] for 1h and washed with PBS. Stained cells were observed under an optical microscope and imaged with a digital camera (Nikon, Tokyo, Japan). To quantify intracellular Oil Red O staining, 100% isopropanol (Merk KGaA, Darmstadt, Germany) was added in stained cell, and absorbance was measured at 510 nm using a Versa Max microplate reader.

### 2.7. Gene Knockdown

Cells were transfected with FMOD, MSTN shRNA, or scrambled vector (1 ng) using transfection reagent and media (Santa Cruz Biotechnology, Santa Cruz, CA, USA) according to the manufacturer’s instructions. Successfully transfected cells were selected with Puromycin (2 µg/mL; Santa Cruz Biotechnology). Transfection efficiencies of the knockdown were confirmed by real time PCR between scrambled and knockdown construct transfected cells.

### 2.8. Giemsa Staining and Fusion Indices

C2C12 cells were washed with PBS, fixed with methanol/PBS, and stained with Giemsa G250 (Sigma Aldrich). Images were obtained at 300x and the numbers of nuclei in myotubes and total nuclei were counted in each image. Fusion indices were calculated by expressing the number of nuclei integrated into myotubes as a percentage of the total number of nuclei.

### 2.9. RNA Extraction, cDNA Synthesis, and Real-Time PCR

Total RNA extraction from cells was performed using Trizol reagent (Thermo Fisher Scientific) according to the manufacturer’s instructions. RNA (2 µg in 20 µL) was used to synthesize the first strand cDNA using random hexamers and reverse transcriptase at 25 °C for 10 min, 37 °C for 120 min, and 85 °C for 5 min. cDNA (2 µL) and gene-specific primers (10 pmol) were used to analyze gene expressions by real-time RT-PCR, which was performed using a 7500 real-time PCR system and power SYBR Green PCR Master Mix (Thermo Fisher Scientific). Primer sequences are provided in Supplementary Table S1.

### 2.10. Western Blot

Total proteins in cells or muscle tissues were extracted using LIPA buffer supplemented with protease inhibitor (Thermo Fisher Scientific) and protein concentrations were measured using the Bradford assay. Proteins (40 µg) were electrophoresed with SDS-polyacrylamide (10 or 12%) gels and transferred to PVDF membranes (EMS–Millipore, Billerica, MA, USA). Membranes were incubated with blocking reagent [3% skim milk or BSA in Tris-buffered saline (TBS)-Tween 20] and overnight with specific primary antibodies [FMOD (1:400), MSTN (1:2000), MYOG (1:500), MuRF1 (1:500), Atrogin1 (1:500), receptor for advanced glycation end-products (RAGE, 1:500), Galactosidase Beta 1 (Glb1, 1:500), peroxisome proliferator- activated receptor gamma (PPARγ, 1:500), CD36 (1:500), CD163 (1:500), or β-actin (1:2000) antibodies (Santa Cruz Biotechnology)] in 1% skim milk or BSA in TBS at 4 °C. After washing, membranes were incubated with horseradish peroxidase (HRP)-conjugated secondary antibody (Santa Cruz Biotechnology) for 1 h at room temperature, and blots were developed using Super Signal West Pico Chemiluminescent Substrate (Thermo Fisher Scientific). Blots were are provided in Supplementary Figure S1.

### 2.11. Immunohistochemistry

Paraffin-embedded muscle sections were deparaffinized using xylene (Junsei, Tokyo, Japan), rehydrated using an ethanol gradient, and treated with methanol/H_2_O_2_ (Junsei) to quench endogenous peroxidase activity. Muscle sections were blocked with 1% normal goat serum (SeraCare Life Sciences, Milford, MA, USA) and incubated with protein-specific antibody [FMOD, MSTN, MYOG, MuRF1, Atrogin1, RAGE, Glb1, PPARγ, CD36, and CD163 (1:50)] overnight at 4 °C. Sections were then treated with HRP-conjugated secondary antibody (1:100; Santa Cruz Biotechnology), incubated for 1 h at room temperature, counterstained with hematoxylin, dehydrated, mounted, and examined under an optical microscope (Leica, Seoul, Korea). Morphological changes were examined in hematoxylin and eosin-stained sections under an optical microscope (Leica, Wetzlar, Germany).

### 2.12. ELISA Analysis

ELISA kit (FMOD: Elabscience, Wuhan, China, MSTN:-R&D Systems NE, Minneapolis, MN, USA) was used to measure FMOD and MSTN protein concentrations. In brief, muscle tissue lysates or plasma were added to specific antibody-coated plates and incubated for 90 min at 37 °C. After removing the upper phase, the antibodies were added and plates were incubated for 1 h at 37 °C. Plates were then washed, enzyme conjugate was added, and plates were incubated for 30 min at 37 °C. Unbound materials were removed by washing, a substrate was added, and the reaction was allowed to proceed for 15 min. After adding stop solution, absorbance was measured at 450 nm using a Versa Max microplate reader.

### 2.13. Statistical Analysis

The significances of differences between the mean of normalized gene expressions were determined using Tukey’s Studentized Range (HSD). GAPDH was used at the internal control, and the analysis was conducted by one-way ANOVA using PROC GLM in SAS ver. 9.0 (SAS Institute, Cary, NC, USA). Image J software (National Institutes of Health, Bethesda, MA, USA, https://imagej.nih.gov/ij/, 1997–2018, accessed on 24 January 2021) was used for the measurement of band intensities in Western blots. Protein expressions were normalized versus β-actin. Values of *p* ≤ 0.05 indicated statistical significance.

## 3. Results

### 3.1. FMOD and MSTN Gene Expressions during Myoblast Proliferation, Differentiation, and Muscle Regeneration

**Figure 1:**
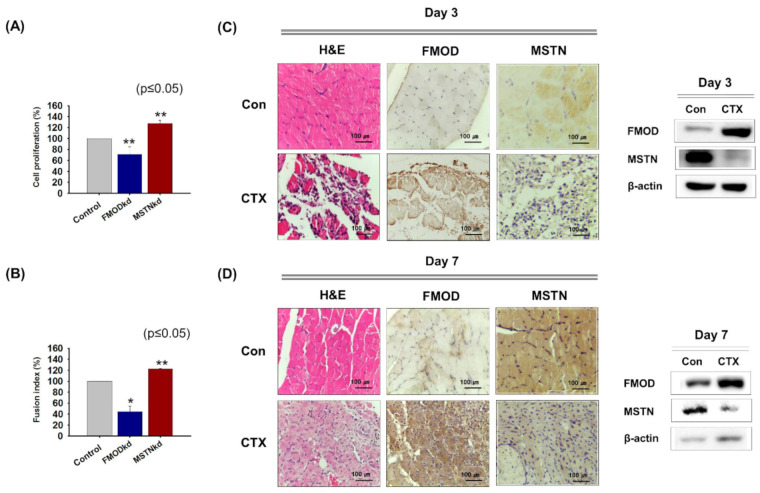
**FMOD and MSTN gene expressions during myoblast proliferation, differentiation, and muscle regeneration.** (**A**) FMOD and MSTN knockdown C2C12 cells were incubated with growth media for 2 days and cell proliferation was assessed using an MTT assay. (**B**) FMOD and MSTN knockdown cells were incubated in differentiation media for 4 days. Cell nuclei were stained with Giemsa solution and then fusion indices were calculated by expressing the number of nuclei integrated into myotube as a percentage of the total number of nuclei. (**C**,**D**) CTX was injected once into gas muscles and mice were sacrificed 3 or 7 days later. Expressions of FMOD and MSTN in control and CT- injected muscles were assessed immunohistochemically and by Western blot. FMODwt and MSTNwt indicate cells transfected with scrambled vector. Means ± SD (n > 3). * *p* ≤ 0.05, *** *p* ≤ 0.001.

FMOD and MSTN were knocked down (FMOD_kd_ or MSTN_kd_) in C2C12 cells and cells were incubated in growth or differentiation media for 2 or 4 days, respectively. Cell proliferation was lower in FMOD_kd_ cells but higher in MSTN_kd_ cells than in wild type controls (Figure 1A). Similarly, during myogenic differentiation, fusion indices were lower in FMOD_kd_ cells but higher in MSTN_kd_ cells than in wild type controls (Figure 1B). In the muscle regeneration study, 3 and 7 days after CTX injection, FMOD protein levels were greater than in non-injected muscles, but MSTN levels were lower (Figure 1C,D). This experiment demonstrated that the expressions of FMOD and MSTN move in opposite directions during myoblast proliferation, differentiation, and muscle regeneration.

### 3.2. Gene Expressions in MSTN Knockout Mice

**Figure 2:**
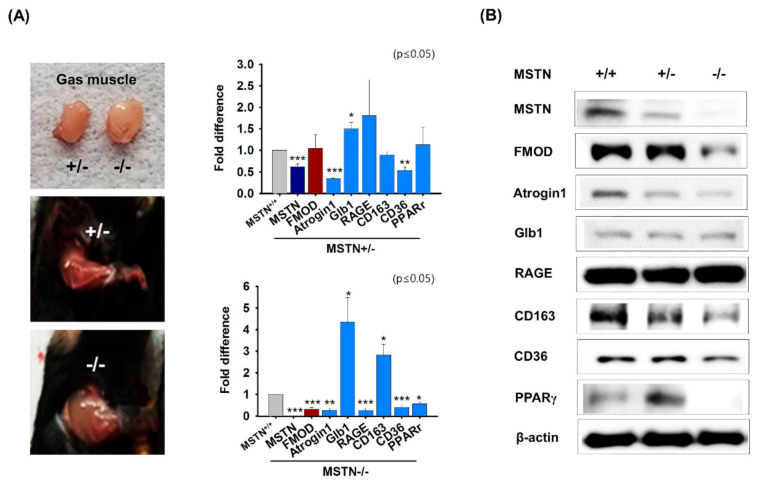
**FMOD and MSTN expressions in MSTN^−/^^−^ mouse muscles.** (**A**,**B**) mRNA and protein expressions were determined by real-time RT-PCR and Western blot in MSTN^+/+^, MSTN^+/^^−^, and MSTN^−/−^ muscles. MSTN^+/+^, MSTN^+/^^−^, and MSTN^−/^^−^ indicates the wild type, heterozygote, and homozygote, respectively. Means ± SD (n > 3). * *p* ≤ 0.05, ** *p* ≤ 0.001, *** *p* ≤ 0.0001.

Muscle aging (Atrogin 1 and Glb1), diabetes (RAGE and CD163), and intracellular lipid accumulation (CD36 and PPARγ) related mRNA and protein expressions and FMOD were analyzed in MSTN knockout gas muscles. Muscle mass was greater in MSTN^−/−^ mice than in MSTN^+/−^ mice. mRNA and protein expressions of FMOD, Atrogin1, CD36, and PPARγ were lower in MSTN^−/−^ gas muscles at the transcriptional and translational levels than in normal muscles, but Glb1 and CD163 mRNA expressions were higher. However, CD163 protein levels were lower, and Glb1 protein levels were unchanged in MSTN^−/−^ muscles (Figure 2A,B). In addition, MSTN knockdown C2C12 cells were incubated in differentiation media for 4 days. The expressions of MSTN, Atrogin1, MYOG, FMOD, CD36, and PPARγ were decreased in MSTN_kd_ cells more than in MSTN_wt_ cells, whereas the levels of CD163, Glb1, and RAGE were increased (Supplementary Figure S2A,B). The results obtained using MSTN knockout mice and knockdown C2C12 cell suggested that mRNA and protein expressions of FMOD, Atrogin 1, CD36, and PPARγ are controlled by MSTN.

### 3.3. Age-Dependent FMOD and MSTN Expressions in Muscles

**Figure 3:**
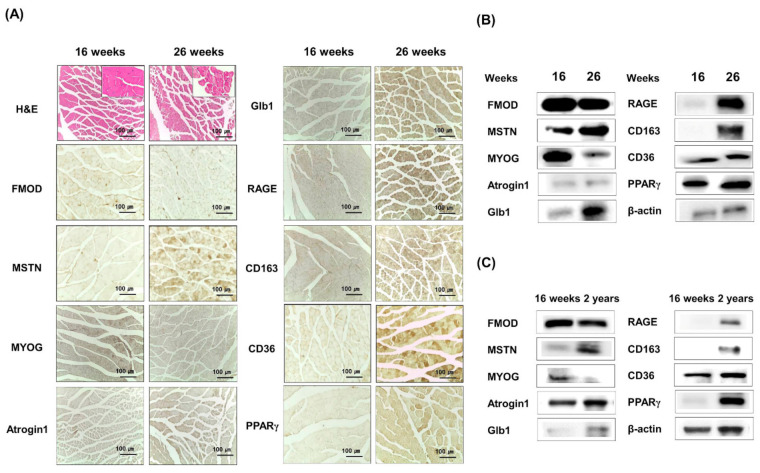
**FMOD and MSTN expressions in the muscles of 16- and 26-week and 2-year-old mouse muscle tissues.** (**A**,**B**) Atrogin1, MYOG, FMOD, MSTN, CD163, Glb1, RAGE, CD36, and PPARγ protein levels were determined immunohistochemically and by Western blot in 16- and 26-week-old gas muscle tissues. (**C**) Atrogin1, MYOG, FMOD, MSTN, CD163, Glb1, RAGE, CD36, and PPARγ levels were determined by Western blot in 16-week and 2-year-old muscle tissues.

FMOD and MSTN protein expressions were analyzed in 16- or 26-week-old mouse muscles. FMOD and MYOG protein levels were lower in 26-week-old than in 16-week-old mice, but protein levels of MSTN, Atrogin 1, CD163, RAGE, Glb1, CD36, and PPARγ tended to increase (Figure 3A,B, Supplementary Figure S3). Furthermore, a comparison of 16-week- and 2-year-old muscles showed FMOD and MYOG protein levels continued to decrease, and those of MSTN, Atrogin 1, CD163, RAGE, Glb1, CD36, and PPARγ continued to increase (Figure 3C), which showed the gene expressions of FMOD and MSTN in muscles move in opposite directions during aging.

### 3.4. FMOD and MSTN Gene Expression in Ceramide Treated Cells

**Figure 4:**
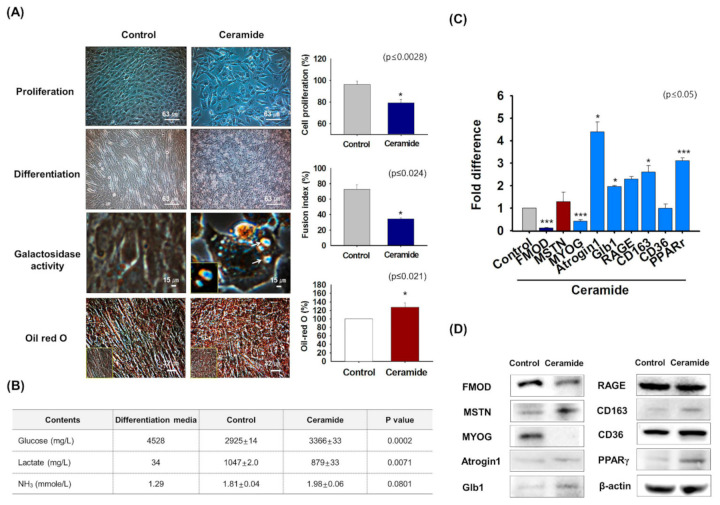
**FMOD and MSTN expressions in ceramide treated cells.** C2C12 cells were cultured for 2 days in proliferation or differentiation medium supplemented with ceramide. (**A**) Cell proliferation and galactosidase activity in growth medium supplemented with ceramide were measured by MTT and X-gal staining, respectively. Myotube formation and lipid accumulation in differentiation media supplemented with ceramide were analyzed by Giemsa and Oil red O staining, respectively. (**B**) Cells were cultured with differentiation media supplemented with ceramide for 2 days. Metabolite (glucose, lactate, and NH_3_) concentrations were measured in control and ceramide-treated cells culture media (**C**,**D**) FMOD, MSTN, MYOG, Atrogin1, Gib1, RAGE, CD163, CD36, and PPARγ mRNA and protein expressions were assessed by real-time RT-PCR and Western blot, respectively. Control indicates non-treated cells. Means ± SD (n > 3). * *p* ≤ 0.05, *** *p* ≤ 0.0001.

Ceramide reduces myoblast proliferation and differentiation [41]. To investigate the functions of FMOD and MSTN in aged cells, C2C12 cells were treated with ceramide for 2 days, and proliferation, myogenic differentiation, β-galactosidase activity, and Oil-red O staining were assessed. Cell proliferation and myotube formation were diminished in ceramide-treated cells, which showed narrower myotubes and higher levels of β-galactosidase and Oil-red O intensity than non-treated controls (Figure 4A). Analysis of metabolites in cell culture media showed ceramide treatment increased glucose and NH_3_ levels and reduced lactate levels as compared with controls (Figure 4B). FMOD, MSTN, MYOG, and CD36 mRNA expression were increased and Atrogin1, Glb1, RAGE, PPARγ, p53, and FOXO3 expressions were decreased in differentiated cells (Day 2) compared with control (Day 0) (Supplementary Figure S4). As observed in aged mice (Figure 3), FMOD levels in myogenic differentiation media were reduced by ceramide, while the levels of MSTN increased and aged-related gene expression was increased in ceramide-treated cells compared with control cells (Figure 4C,D, Supplementary Figure S5). Interestingly, these results showed that the results obtained by treating C2C12 cells with ceramide agreed well with the observed effects of aging.

### 3.5. Effect of Adipogenic Conditions on FMOD and MSTN Gene Expressions in Myoblasts

**Figure 5:**
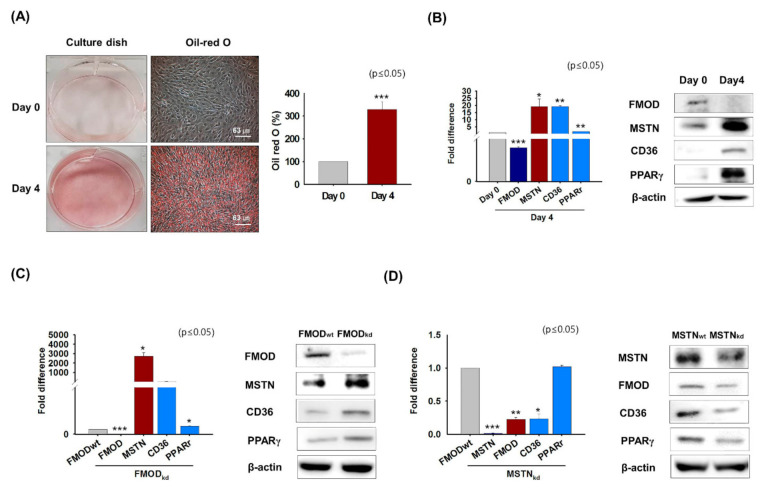
**FMOD and MSTN expressions under adipogenic conditions.** C2C12 cells were incubated in adipogenic media for 4 days. (**A**) Oil-red O staining was performed and intensities were measured at day 0 and after culture for 4 days. (**B**) mRNA and protein expressions of FMOD, MSTN, CD36, and PPARγ were determined by real-time RT-PCR and Western blot, respectively. (**C**,**D**) FMOD and MSTN were knocked down and cells were incubated in adipogenic media for 4 days**.** mRNA and protein levels of FMOD, MSTN, CD36, and PPAR were determined by real-time RT-PCR and Western blot, respectively. FMOD_wt_ and MSTN_wt_ indicate cells transfected with scrambled vector. Means ± SD (n > 3). * *p* ≤ 0.05, ** *p* ≤ 0.001, *** *p* ≤ 0.0001.

Previous studies have shown that intracellular fat levels are elevated in aged muscle [46] and that FMOD inhibits lipid accumulation by negatively regulating the expressions of CD36 and PPARγ during myogenesis [4]. Thus, this experiment was conducted to create conditions similar to those in aged muscles in order to observe changes in the expressions of genes involved in fatty acid uptake (CD36) and lipid metabolism (PPARγ) and those of the FMOD and MSTN genes. C2C12 cells stained with Oil red O solution after incubation in adipogenic medium for 4 days had higher Oil-red O intensities than day 0 (Figure 5A). Under adipogenic conditions, the mRNA and protein expression of FMOD decreased and those of MSTN, CD36, and PPARγ increased (Figure 5B). When FMOD or MSTN knockdown cells were cultured in adipogenic medium for 4 days, the mRNA and protein expressions of MSTN, CD36, and PPARγ increased in FMOD_kd_ cells, whereas the mRNA and protein expressions of FMOD, CD36, and PPARγ decreased in MSTN_kd_ cells (Figure 5C,D). These results show that under adipogenic condition the expressions of genes involved in intracellular fat accumulation increase and the expressions of genes involved in myogenesis decrease. Furthermore, they suggest that FMOD negatively regulates genes involved in intracellular fat accumulation and that MSTN positively regulates these genes.

### 3.6. FMOD and MSTN Gene Expressions in High-Fat Diet Mice

**Figure 6:**
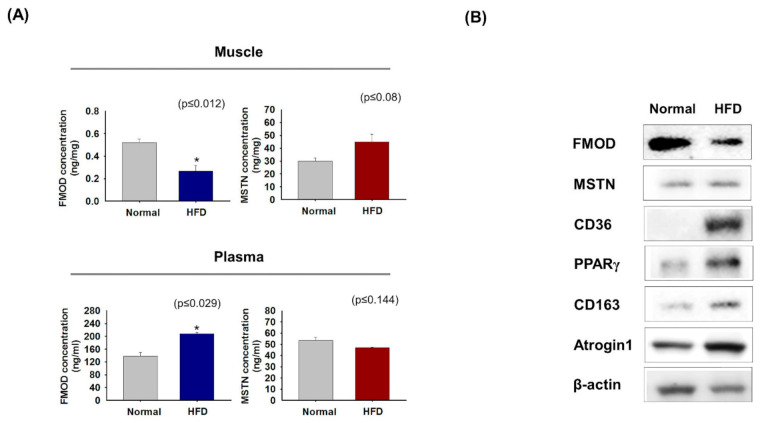
**FMOD and MSTN protein levels in normal and HFD mouse muscles and plasma.** (**A**) FMOD and MSTN protein concentrations were assessed by ELISA in normal and HFD mice gas muscles and plasma. (**B**) FMOD, MSTN, CD36, PPARγ, CD163, and Atrogin1 protein levels were determined by Western blot. Normal indicates muscle and plasma of mice fed a normal diet. Means ± SD (n > 3). * *p* ≤ 0.05.

FMOD and MSTN protein levels in mice raised on a normal or high-fat diet (HFD) were measured in gas muscle and plasma. HFD-fed mice had lower FMOD levels in gas muscles and higher levels in plasma than mice fed a normal diet, whereas MSTN levels were higher in muscle and similar in plasma (Figure 6A). FMOD protein levels were lower in HFD mice muscle than in controls, but MSTN, CD36, PPARγ, CD163, and Atrogin 1 levels were higher (Figure 6B), which is similar to that observed in aged mice (Figure 3) and ceramide-treated C2C12 cells (Figure 4).

## 4. Discussion

MSTN is a chalone like molecule that controls SM mass [47,48]. MSTN inhibition increases muscle mass in mice [49], and muscle fiber counts have been reported to be greater in MSTN knockout (MSTN^−/−^) mice during development [45,50]. Furthermore, MSTN^−/−^ mice have been reported to have more muscle mass, less fat mass, and more resistance to diet and genetically induced obesity [17], and a decrease in the role and number of MSCs during aging was reported to result in loss of nuclei in large fibers [51]. Muscle homeostasis is normally regulated by MSTN and its upregulation has been associated with SM degeneration and the muscle wasting of cachexia associated with cancer and sarcopenia [52,53].

In the previous study, we found FMOD promoted myogenesis by interacting with MSTN protein to prevent MSTN binding to ACVRIIB and by inhibiting the transcription of MSTN [26]. Since MSTN is a well-known negative regulator of muscle growth, and FMOD is a novel regulator of MSTN, we aimed to determine how these two proteins interact during myogenesis in aged muscles. We found FMOD negatively regulated MSTN during myoblast proliferation and differentiation and that the expression patterns of FMOD and MSTN moved in opposite directions, that is, FMOD expression increased and MSTN expression decreased, during myogenesis. In our mouse model studies of muscle regeneration (Figure 1), MSTN knockout (Figure 2), muscle aging (Figure 3), and HFD treatment (Figure 6), the expression patterns of FMOD and MSTN genes were always found to be opposite. Furthermore, similar results were obtained in our in vitro model of muscle aging, when C2C12 cells were treated with ceramide during differentiation (Figure 4) as well as under adipogenic conditions (Figure 5). In addition, a significant reduction in FMOD gene expression was observed in MSTN^−/−^ mice (Figure 2) and in MSTN_kd_ C2C12 cells during differentiation (Supplementary Figure S2), suggesting that the FMOD gene is positively regulated by MSTN. Hence, the present study shows that FMOD negatively regulates MSTN gene expression, but MSTN positively regulates FMOD gene expression.

SM aging manifests as a loss of muscle volume and function. Several factors (e.g., nutritional, hormonal, metabolic, neurological, and immunological) directly or indirectly affect this aging-associated phenotype and lead to sarcopenia [54]. The absence of MSTN in fat tissues is associated with elevated peripheral tissue fatty acid oxidation, enhanced mitochondrial function, and decreased adipose tissue mass. Furthermore, siRNA knockdown of MSTN reduces visceral fat levels in adult mice [55,56]. The introduction of MSTN protein-like molecules and the manipulation of endogenous MSTN levels also offer potential means for preventing or treating metabolic ailments like muscle wasting, type 2 diabetes, and obesity [57]. Decorin, follistatin (FST), FST-like related gene (FLRG), and GASP-1 and GASP-2 (growth and differentiation factor-associated serum protein-1 and -2) have also been reported to act as MSTN antagonists and enhance the differentiation and proliferation rate of myogenic cells [58,59,60,61].

**Figure 7:**
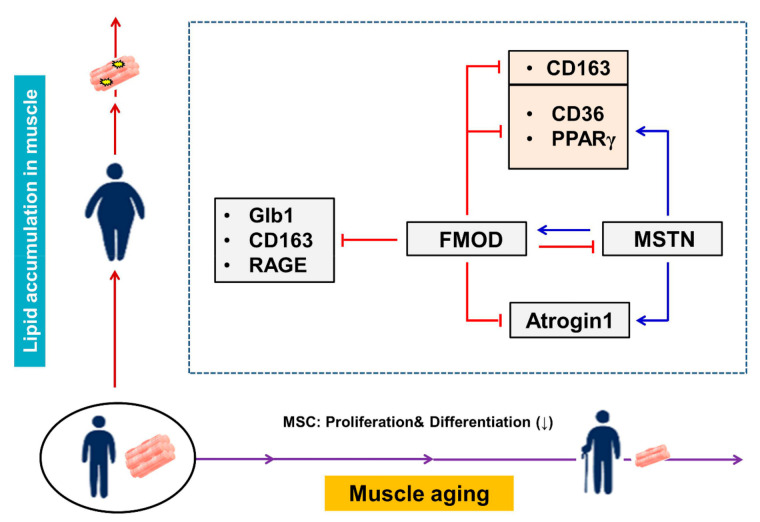
**Summary of proposed relationships between the expressions of FMOD and MSTN during aging.** The inverse relationship between MSTN and FMOD. FMOD inhibits Glb1, CD163, RAGE, and Atrogin1. MSTN increases Atrogin1 expression. FMOD negatively regulates the expressions of CD36, PPARγ, and CD163.

As mentioned above, muscle aging is associated with diabetes and obesity, and in the present study, we compared the expressions of the FMOD and MSTN genes and those of genes related to muscle aging (Atrogin 1, Glb1), diabetes (RAGE, CD163) and intracellular lipid accumulation (CD36, PPARγ) in vivo and in vitro. Interestingly, FMOD gene expression was diminished in aged and HFD mouse muscles and in C2C12 cells cultured in the presence of ceramide or under adipogenic conditions during differentiation, whereas the expressions of genes related to aging, diabetes, and obesity and that of MSTN were all elevated. In addition, the expressions of genes related to muscle aging (Atrogin 1) and intracellular lipid accumulation (CD36, PPARγ) were reduced in MSTN^−/−^ mice. These results imply that the expressions of these six genes associated with muscle aging, diabetes, and intracellular lipid accumulation are negatively regulated by FMOD through the direct inhibition or indirect inhibition of MSTN, and that the expressions of the Atrogin1, CD36, and PPARγ genes are positively regulated by MSTN (Figure 7). Furthermore, these suggestions raise two questions, that is, how does MSTN regulate these six genes? and how does FMOD upregulate MSTN?

Thus, our findings indicate that MSTN positively regulates the expressions of muscle aging and intracellular lipid accumulation genes in muscles, as evidenced by an increase in muscle mass in MSTN^−/−^ mice. These findings concur with those of previous studies [45,62] and with another study, in which MSTN^−/−^ mice had higher muscle masses, lower fat masses, and exhibited resistance to diet-induced and genetic obesity [17]. Here, we report that FMOD and MYOG expressions were higher in the muscles of 16-week-old than in those of 2-year-old mice; however, expressions of muscle aging, diabetes, and intracellular lipid accumulation-related genes were higher in aged mice (2-year-old) muscles (Figure 3). In addition, decreased FMOD and MYOG expression with increased Atrogin1, RAGE, Glb1, CD163, CD36, and PPARγ expressions were found in FMOD_kd_ cells relative to the FMOD_wt_ cells (Supplementary Figure S2C,D). It was reported that the level of MSTN mRNA in the gastrocnemius declined with age, being 9, 34, and 56% lower at 6, 12, and 27 months, respectively [63].

Myoblast proliferation and differentiation were suppressed in ceramide-treated cells as compared with non-treated controls. High lipid and ceramide concentrations are markers in muscles in obese subjects [64]. We found myoblasts treated with ceramide stained more intensely for β-galactosidase (Figure 4) and that ceramide at high concentrations reduced myoblast proliferation, disrupted cell cycle regulation, and induced a senescent phenotype, which adds to the understanding of SM cell adaptation during conditions of high intramuscular lipid deposition and/or obesity [65]. Interestingly, FMOD expression was lower but CD36 and MSTN expressions were higher in ceramide-treated cells than in controls (Figure 4C,D). Ceramides stimulate insulin resistance in several metabolically active tissues including SM, which is the main site of insulin-stimulated glucose disposal [66]. Interestingly, ceramide treatment upregulates insulin-resistant gene (CD163 gene) level i.e., aged cells are unable to utilize the glucose, as its metabolism is an important regulatory parameter for the aging process. We investigated the levels of glucose, lactate and NH_3_ metabolites in the culture media of control and ceramide-treated C2C12 cells (Figure 4B). Glucose levels were higher in the conditioned media of ceramide-treated cells than in those of controls, whereas lactate levels were lower. Ammonia levels were slightly higher in the conditioned media of ceramide-treated cells. These observations suggest ceramide inhibits glucose uptake, increases NH_3_ release, but reduces lactate production. It has been previously reported that ammonia level in C2C12 cell was elevated in muscle wasting and that this increased MSTN expression and decreased myotube diameters [67].

C2C12 cells were cultured under adipogenic conditions to investigate the relationship between FMOD and MSTN during intracellular lipid accumulation (Figure 5). Oil-red O intensities were higher in cells cultured under adipogenic conditions for 4 days. Furthermore, CD36 and PPARγ expressions were elevated in FMOD_kd_ cells but depressed in MSTN_kd_ cells. PPARγ regulates CD36 expression by virtue of a PPARγ responsive element in the proximal region of CD36 promoter [68], and CD36 deficiency reduces fatty acid uptake and may lead to insulin resistance, which indicates CD36 is metabolically protective in adipose and muscle tissues [69]. We observed FMOD protein concentrations were lower in the gas muscles of HFD-fed mice than in normal controls, which suggests FMOD plays an important inhibition role in lipid metabolism and adipogenesis (Figure 6).

Future studies are needed to elucidate the direct mechanism between FMOD and MSTN during human muscle aging for the management of sarcopenia.

## 5. Conclusions

The present study indicates MSTN promotes muscle aging by upregulating the expressions of genes related to muscle aging (Atrogin1) and intracellular lipid accumulation (CD36, PPARγ) in muscles. On the other hand, it suggests FMOD inhibits aging by downregulating genes involved in muscle aging, diabetes, and intracellular lipid accumulation or by indirectly inhibiting their expressions by suppressing the activity of MSTN protein.

## Data Availability

The data presented in this study are available in this article and the accompanying supplementary material.

## Supplementary Materials

The following are available online at https://www.mdpi.com/article/10.3390/cells10082083/s1, Table S1: Primers information, Figure S1: Western whole blots, Figure S2: Gene expressions in FMOD and MSTN knockdown cells. FMOD and MSTN knockdown cells were incubated in differentiation media for 4 days. (A&B) Gene expressions of FMOD, MSTN, MYOG, Atrogin1, Glb1, RAGE, CD163, CD36, and PPARγ in MSTN_kd_ and (C&D) FMOD_kd_ cells were determined by real-time RT-PCR and Western blot, respectively. FMOD_wt_ and MSTN_wt_ indicate cells transfected with scrambled vector. Means ± SD (n > 3). * *p* ≤ 0.05, ** *p* ≤ 0.001, *** *p* ≤ 0.0001, Figure S3: Protein expression between 16 and 26 weeks old muscles. Protein expression was determined by Western blot and band intensities were measured by Image J, Figure S4. mRNA expressions in 0 and 2 days differentiated cells. C2C12 cells were cultured with differentiation media for 2 days; FMOD, MSTN, MYOG, CD36, Myosin heavy chain (MYH), Atrogin1, Glb1, RAGE, PPARγ, p53, and FOXO3 mRNA expressions were analyzed by real-time RT-PCR. Means ± SD (n > 3). * *p* ≤ 0.05, ** *p* ≤ 0.001, *** *p* ≤ 0.0001. Figure S5: p53, Bax and FOXO3 genes expression in ceramide-treated cells. C2C12 cells were cultured with differentiation media supplemented with 50 uM ceramide for 2 days, and p53, Bax and FOXO3 mRNA expression were analyzed by real-time RT-PCR. Means ± SD (n > 3). * *p* ≤ 0.05, ** *p* ≤ 0.001, *** *p* ≤ 0.0001.

## Abbreviations

SM	Skeletal muscle
MSCs	Muscle stem cells
MYOG	Myogenin
MSTN	Myostatin
TGF-β	Transforming growth factor-β
FMOD	Fibromodulin
ECM	Extracellular matrix
CTX	Cardiotoxin
PPARγ	Peroxisome proliferator-activated receptor gamma
Glb1	Galactosidase Beta 1
ECM	Extracellular matrix
Gas muscle	Gastrocnemius muscle
HFD	High-fat diet
ACVRIIB	Type IIB activin receptor
MRF4	Muscle regulatory factor 4
IMCL	Intramyocellular lipid
AGE	Advanced glycation end product
FST	Follistatin
FLRG	FST-like related gene
GASP-1	Growth and differentiation factor-associated serum protein-1
MYH	Myosin heavy chain
RAGE	Receptor for advanced glycation end-products
GASP-2	Growth and differentiation factor-associated serum protein-2
