# Supplementary material for: Interaction of Fibromodulin and Myostatin to Regulate Skeletal Muscle Aging: An Opposite Regulation in Muscle Aging, Diabetes, and Intracellular Lipid Accumulation
Source: Cells. 2021 Aug 13;10(8):2083. doi: 10.3390/cells10082083 (PMC8393414; doi:10.3390/cells10082083)

Supplementary Table S1. Primer information

Species	Gene	Product size (bp)	Tm (°C)	Sequence (F)	Sequence (R)
Mouse	GAPDH	155	59	5'-tgctggtgctgagtatgtcg-3'	5'-caagcagttggtggtacagg-3'
Mouse	MSTN	163	59	5'-acgctaccacggaaacaatc-3'	5'-ggagtcttgacgggtctgag-3'
Mouse	MYOG	185	59	5'-tccagtacattgagcgccta-3'	5'-caaatgatctcctgggttgg-3'
Mouse	MYL2	177	59	5'-aaagaggctccaggtccaat-3'	5'-cctctctgcttgtgtggtca-3'
Mouse	Atrogin1	160	59	5'-ttcagcagcctgaactacga-3'	5'-tgaaagcttcccccaaagta-3'
Mouse	CD163	218	59	5'-ctggtcgtgtggaagtgaaa-3'	5'-cgccactgagcatagtgaaa-3'
Mouse	RAGE	160	59	5'-aggaacgtgcagagctgaat-3'	5'-ctggttggagaaggaagtgc-3'
Mouse	Glb1	228	59	5'-cctattggaatggtgccaac-3'	5'-tacccagagcttcagccact-3'
Mouse	PPARγ	232	59	5'-aagagctgacccaatggttg-3'	5'-acccttgcatccttcacaag-3'
Mouse	CD36	187	59	5'-tggagctgttattggtgcag-3'	5'-tgggttttgcacatcaaaga-3'
Mouse	P53	242	59	5'-cgggtggaaggaaatttgta-3'	5'-gcacaaacacgaacctcaaa-3'
Mouse	Bax	173	59	5'-tgcagaggatgattgctgac-3'	5'-gatcagctcgggcactttag-3'
Mouse	FOXO3	215	59	5'-ttcaaggataagggcgacag-3'	5'-cctcggctcttggtgtactt-3'

Supplementary Figure S1.

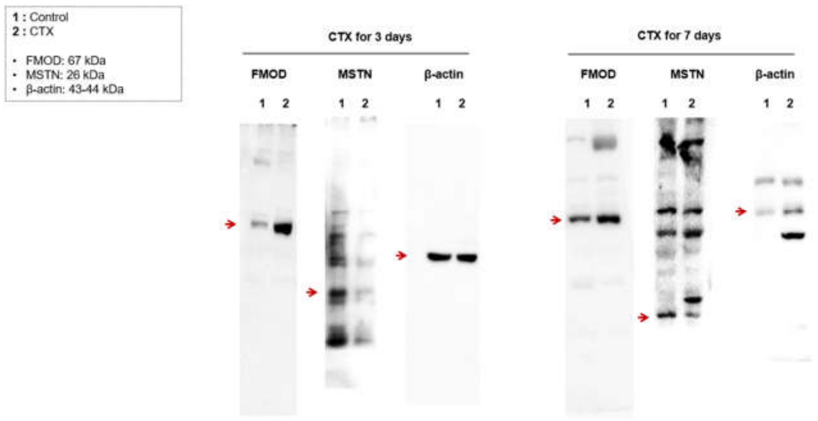

Figure 2

1 : MSTN +/+, **2:** MSTN +/-, **3:** MSTN-/-

- MSTN: 26 kDa, FMOD: 67 kDa, Atrogin1: 42 kDa, Glb1: 76kDa, RAGE: 46 kDa, CD163: 130 kDa, CD36: 88-90kDa, PPARγ: 54-57kDa,β-actin : 43-44 kDa

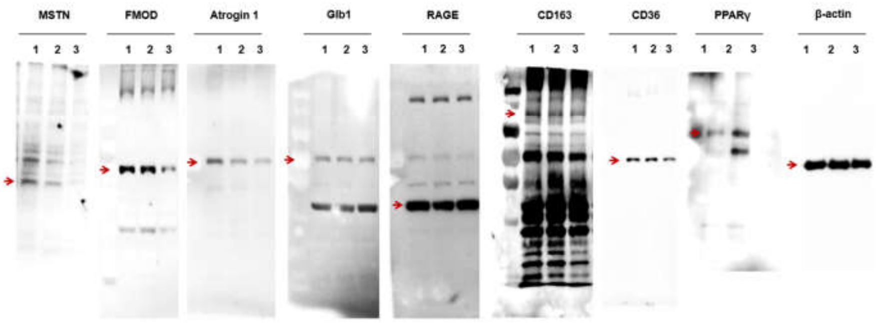

Figure 3

1 : 16 weeks muscle, 2: 26 weeks muscle

- FMOD: 67 kDa, MSTN: 67 kDa, Atrogin1: 42 kDa, Glb1: 76kDa, RAGE: 46 kDa, CD163: 130 kDa, CD36: 88-90kDa, PPARγ: 54-57kDa,β-actin : 43-44 kDa

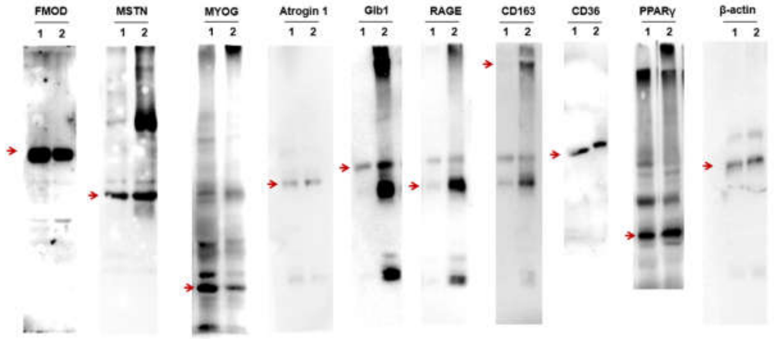

Figure 3

1 : 16 weeks muscle, 2: 2 years muscle

- FMOD: 67 kDa, MSTN: 67 kDa, Atrogin1: 42 kDa, Glb1: 76kDa, RAGE: 46 kDa, CD163: 130 kDa, CD36: 88-90kDa, PPARγ: 54-57kDa ,β-actin : 43-44 kDa

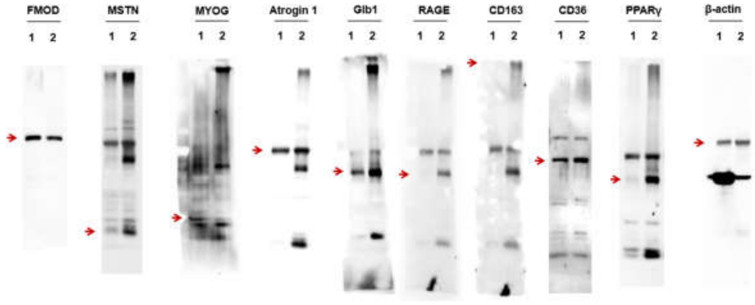

Figure 4

1 : Control, 2: Ceramide

- FMOD: 67 kDa, MSTN: 67 kDa, Atrogin1: 42 kDa, Glb1: 76kDa, RAGE: 46 kDa, CD163: 130 kDa, CD36: 88-90kDa, PPARγ: 54-57kDa,β-actin : 43-44 kDa

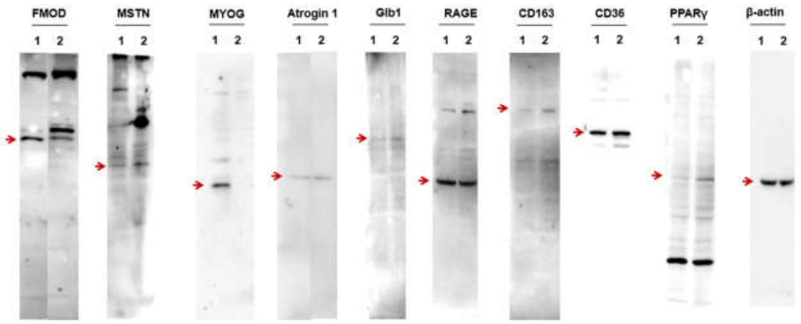

Figure 5

1 : Day 0, 2: Day 4

- FMOD: 67 kDa, MSTN: 67 kDa, CD36: 88-90kDa, PPARγ:54-57kDa,β-actin:43-44 kDa

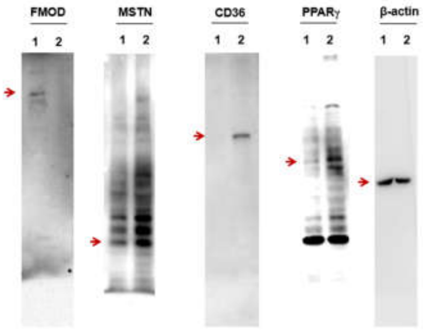

Figure 5

1 : FMODwt, 2. FMODkd

- FMOD: 67 kDa, MSTN: 67 kDa, CD36: 88-90kDa, PPARγ:54-57kDa,β-actin:43-44 kDa

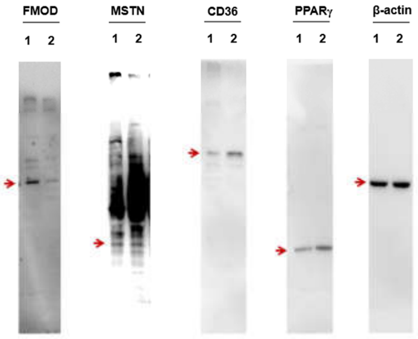

Figure 5

1 : MSTNwt, 2. MSTNkd

- FMOD: 67 kDa, MSTN: 67 kDa, CD36: 88-90kDa, PPARγ:54-57kDa,β-actin:43-44 kDa

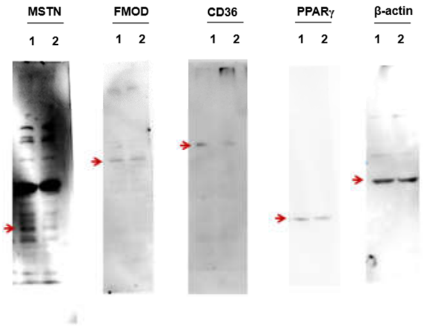

Figure 6

1 : Normal, 2. HFD

- FMOD: 67 kDa, MSTN: 67 kDa, CD36: 88-90kDa, PPARγ:54-57kDa,β-actin:43-44 kDa, CD163: 130 kDa, Atrogin1: 42 kDa

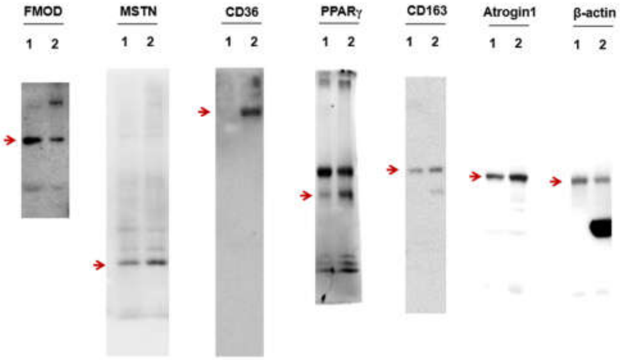

Supplementary Figure 1

1 : MSTNwt, 2. MSTNkd

- FMOD: 67 kDa, MSTN: 67 kDa, CD36: 88-90kDa, PPARγ:54-57kDa,β-actin:43-44 kDa, CD163: 130 kDa, Atrogin1: 42 kDa

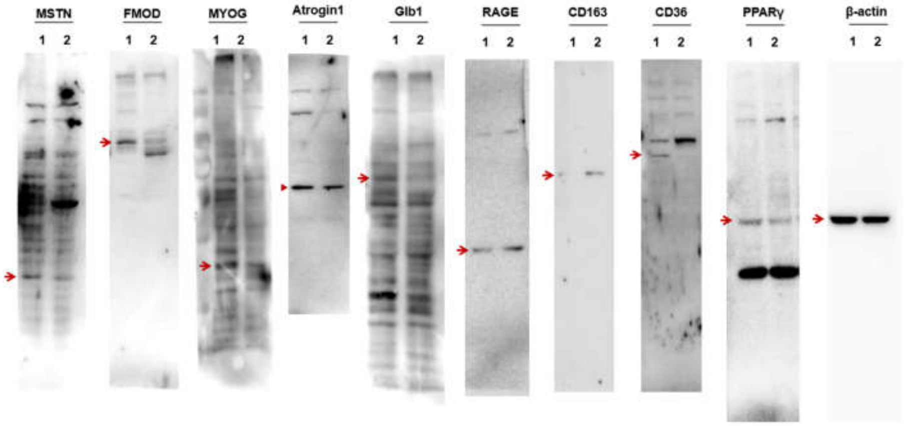

Supplementary Figure 1

FMODwt_2%FBS

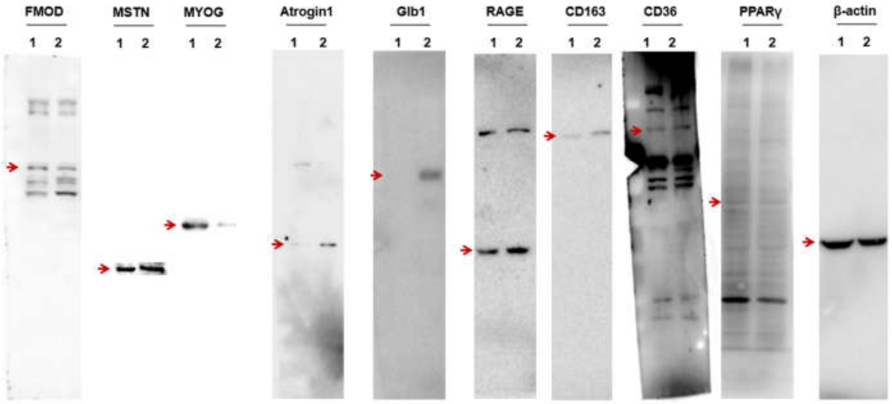

Supplementary Figure S2.

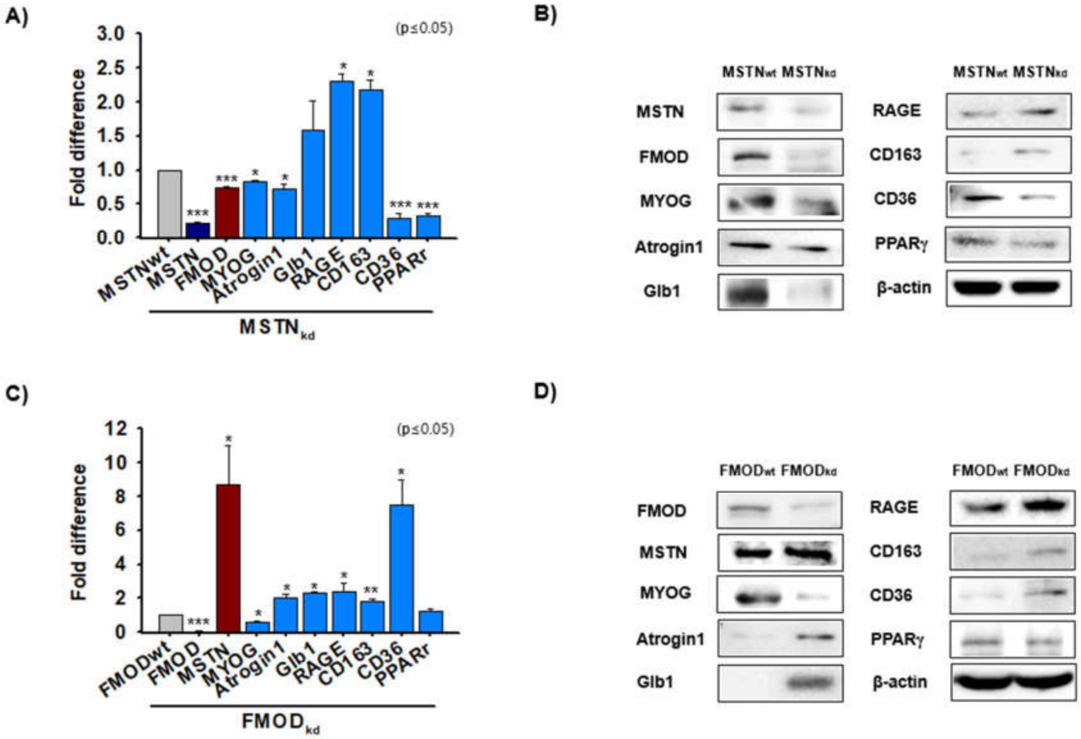

Supplementary Figure S3.

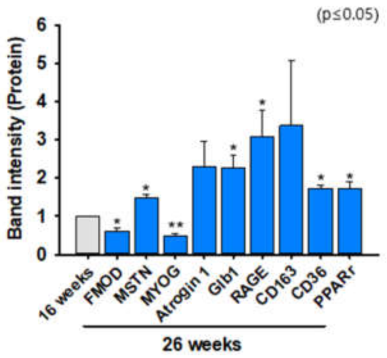

Supplementary Figure S4.

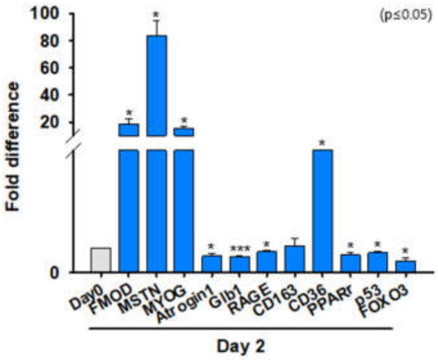

Supplementary Figure S5.

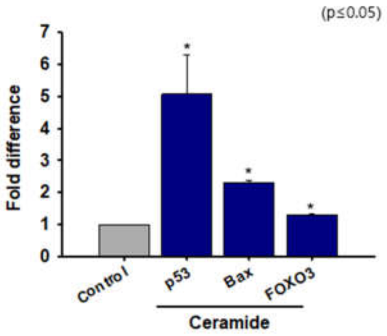

Supplement: Supplementary file 1 [file cells-10-02083-s001.zip › cells-1310834-supplementary.pdf]
